# Synthesis and biological evaluation of certain hydrazonoindolin-2-one derivatives as new potent anti-proliferative agents

**DOI:** 10.1080/14756366.2018.1462802

**Published:** 2018-04-30

**Authors:** Wagdy M. Eldehna, Reem I. Al-Wabli, Maha S. Almutairi, Adam B. Keeton, Gary A. Piazza, Hatem A. Abdel-Aziz, Mohamed I. Attia

**Affiliations:** a Department of Pharmaceutical Chemistry, Faculty of Pharmacy, Kafrelsheikh University, Kafrelsheikh, Egypt;; b Department of Pharmaceutical Chemistry, College of Pharmacy, King Saud University, Riyadh, Saudi Arabia;; c Department of Oncologic Sciences and Pharmacology, Drug Discovery Research Center, Mitchell Cancer Institute, University of South Alabama, Mobile, AL, USA;; d Department of Applied Organic Chemistry, National Research Centre, Giza, Egypt;; e Medicinal and Pharmaceutical Chemistry Department, Pharmaceutical and Drug Industries Research Division, National Research Centre (ID: 60014618), Giza, Egypt

**Keywords:** Synthesis, indolin-2-one, retinoblastoma (Rb) protein, multidrug resistant cancer cell lines, cell cycle progression

## Abstract

In connection with our research program on the development of novel indolin-2-one-based anticancer candidates, herein we report the design and synthesis of different series of hydrazonoindolin-2-ones **3a-e**, **5a-e**, **7a-c**, and **10a-l**. The synthesised derivatives were *in vitro* evaluated for their anti-proliferative activity towards lung A-549, colon HT-29, and breast ZR-75 human cancer cell lines. Compounds **5b**, **5c**, **7b**, and **10e** emerged as the most potent derivatives with average IC_50_ values of 4.37, 2.53, 2.14, and 4.66 µM, respectively, which are superior to Sunitinib (average IC_50_ = 8.11 µM). Furthermore, compounds **7b** and **10e** were evaluated for their effects on cell cycle progression and levels of phosphorylated retinoblastoma (Rb) protein in the A-549 cancer cell line. Moreover, **7b** and **10e** inhibited the cell growth of the multidrug-resistant lung cancer NCI-H69AR cell line with IC_50_ = 16 µM. In addition, the cytotoxic activities of **7b** and **10e** were assessed towards three non-tumorigenic cell lines (Intestine IEC-6, Breast MCF-10A, and Fibroblast Swiss-3t3) where both compounds displayed mean tumor selectivity index (1.6 and 1.8) higher than that of Sunitinib (1.4).

## Graphical Abstract

**Figure:**
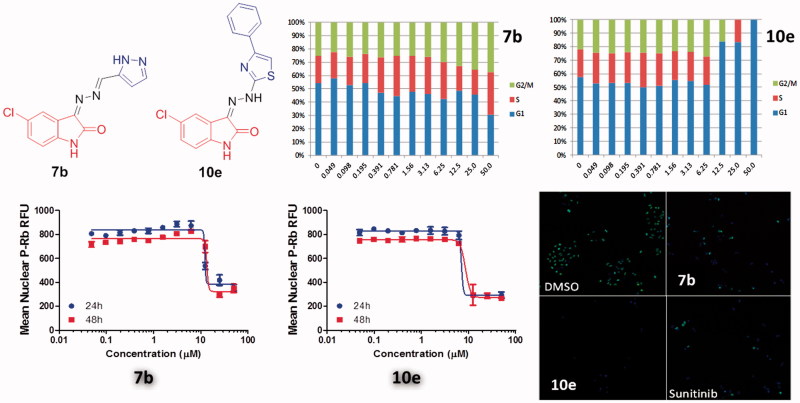

## Introduction

Cancer is a major health burden worldwide and it is deemed to be the second leading cause of mankind mortality after cardiovascular diseases. Most of the clinically available anticancer chemotherapeutic agents are not able to discriminate between cancer cells and the rapidly dividing healthy cells^1^. Moreover, the growing increase in drug resistance and undesired side effects of the clinically available cancer chemotherapeutic agents aroused the necessity to search for newer more potent and safer cancer chemotherapeutic candidates^2^^,^^3^. Subsequently, there is an urgent need to pay much attention to modify and update drug leads from the point of view of medicinal chemistry and drug design to fulfill more effective and potent anticancer agents.

**Figure 1.**
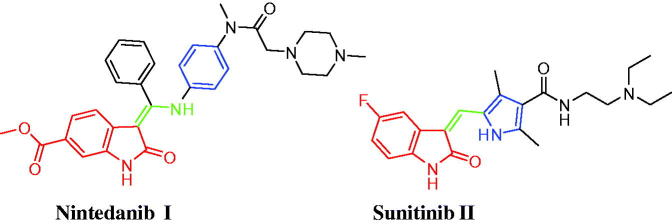
Structures of the isatin-based approved anticancer drugs; Nintedanib **I** and Sunitinib **II**.

Isatin (1*H*-indole-2,3-dione) is a privileged scaffold that is present endogenously in both human and other mammalian fluids and tissues, in addition to its presence in diverse naturally occurring components such as marine natural products and alkaloids^4^. Isatin constitutes a leading class of heterocycles that is endowed with interesting biological activities^4–8^, chiefly anticancer activity. Nintedanib (Ofev^®^) **I** (Figure 1), an orally available triple angiokinase inhibitor, is approved in Europe in combination with docetaxel for patients with advanced non-small cell lung cancer of adenocarcinoma who have progressed to first-line chemotherapy^9^. Currently, Nintedanib is being investigated in patients with various solid tumors including phase III studies in colorectal cancer^10^^,^^11^ and phase I studies in breast cancer^12^. Sunitinib (Sutent^®^) **II** (Figure 1), a 5-fluoro-3-substituted isatin derivative, has been approved by the FDA for the treatment of gastrointestinal stromal tumors (GIST) and advanced renal cell carcinoma (RCC)^13^. It is a multikinase inhibitor that targets VEGFR-1, VEGFR-2, PDGFRb, and c-Kit.

**Figure 2.**
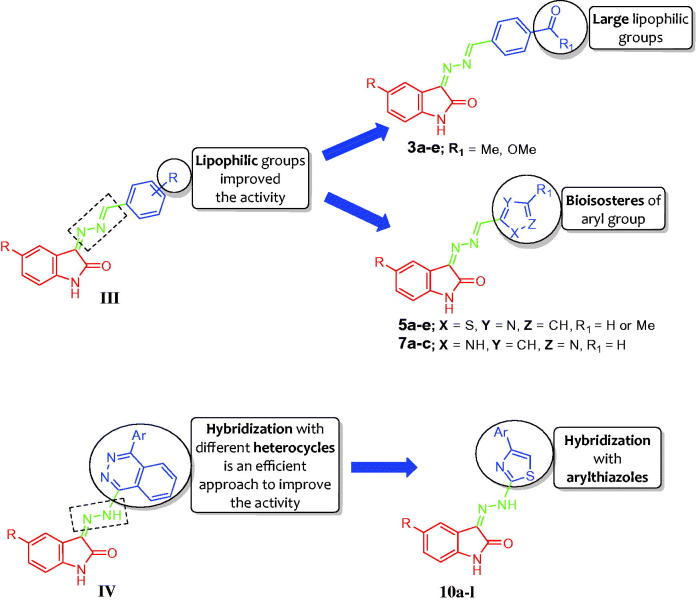
Structures of some reported hydrazonoindoline-2-ones **III** & **IV**, and the target derivatives **3a-e**, **5a-e**, **7a-c**, and **10a-l**.

Recently, our research group has paid much attention to develop many novel small molecules based on the indolin-2-one core as potent anticancer agents^14–22^. In 2017, we reported two studies^14^^,^^15^ regarding development of hydrazonoindolin-2-one-based derivatives and evaluation of their anti-proliferative activity against A549 (lung), HT-29 (colon), and ZR-75 (breast) human cancer cell lines, beside evaluation of their pro-apoptotic activity. In the first study^14^, a series of hydrazonoindolin-2-ones were synthesized with general structure of **III** [3-((-benzylidene)hydrazono)indolin-2-one] (Figure 2). The SAR study concerning compounds **III** suggested that incorporation of lipophilic groups, as methoxy groups, enhances the anti-proliferative activity. Regarding the second study^15^, we adopted the molecular hybridisation approach to design and synthesise four different sets of isatin-quinazoline, isatin-phthalazine, and isatin-quinoxaline hybrids, where phthalazine hybrids **IV** (Figure 2) emerged as the most active anti-proliferative series in this study.

Based on the aforementioned findings and in connection with our research program on the development of indolin-2-one-based anticancer candidates^14–22^, it was thought worthwhile to extend our investigations to probe certain hydrazonoindolin-2-ones displaying promising anti-proliferative activity. In this study, we reported the design and synthesis of different series of hydrazonoindolin-2-ones **3a-e**, **5a-e**, **7a-c**, and **10a-l**, adopting three distinctive strategies to develop such derivatives (Figure 2). The first one focused on grafting of large lipophilic ester or acetyl groups on the pendant phenyl ring of lead structure **III** to furnish target compounds **3a-e** (Figure 2). In the second strategy, a bioisosteric tactic was utilised to replace the pendant substituted phenyl ring in compounds **3a-e** with 2-thiazolyl ring, as in compounds **5a-e**, or 5-pyrazolyl ring, as in compounds **7a-c** (Figure 2), to carry out further elaboration of the hydrazonoindolin-2-ones scaffold and to investigate a valuable SAR. Finally, the hybridisation approach was adopted to conjugate 4-arylthiazoles with indolin-2-one moiety, compounds **10a-l** (Figure 2).

The synthesised hydrazonoindolin-2-ones (**3a-e**, **5a-e**, **7a-c**, and **10a-l**) were *in vitro* evaluated for their anti-proliferative activity towards three human cancer cell lines, namely lung cancer A-549, human colon cancer HT-29, and breast cancer ZR-75 cell lines. Furthermore, the most active anti-proliferative congeners were further evaluated for their effects on cell cycle progression and levels of phosphorylated retinoblastoma (Rb) protein in the A-549 cancer cells. In addition, their effect on multidrug-resistant lung cancer NCI-H69AR cell line was assessed. Finally, the cytotoxic activities of the active members were assessed towards three non-tumorigenic cell lines (Intestine IEC-6, Breast MCF-10A, and Fibroblast Swiss 3t3) to estimate their selectivity for tumor cells.

## Materials and methods

### Chemistry

Melting points were measured with a Stuart melting point apparatus (Bibby Scientific Limited, Staffordshire, UK) and were uncorrected. Mass spectra were recorded using Agilent Quadrupole 6120 LC/MS with ESI (Electrospray ionisation) source (Agilent Technologies, Palo Alto, CA). NMR spectra were recorded on a Bruker NMR spectrometer (Bruker, Karlsruhe, Germany). ^1^H spectra were run at 500 MHz and ^13^C spectra were run at 125 MHz in deuterated dimethyl sulfoxide (DMSO-d_6_). Chemical shifts are expressed in values *δ* (ppm) using the solvent peak as internal standard. All coupling constant (*J*) values are given in Hertz. The abbreviations used are: “s” for singlet, “d” for doublet, and “m” for multiplet. Elemental analyses were carried out at the Regional Center for Microbiology and Biotechnology, Al-Azhar University, Cairo, Egypt. Analytical thin layer chromatography (TLC) on silica gel plates containing UV indicator was employed routinely to follow the course of reactions and to check the purity of products. All reagents and solvents were purified and dried by standard techniques.

#### General procedure for the synthesis of hydrazones 3a-e, 5a-e, and 7a-c

A mixture of equimolar quantities of previously reported hydrazones **1a-c**^19^ (2 mmol) and aldehydes **2a**,**b**, **4a**,**b**, or **6** (2 mmol) in methyl alcohol (30 ml) containing catalytic amount of glacial acetic acid was refluxed for 4 h, and then cooled to room temperature. The precipitates formed were collected by filtration, dried and crystallised from EtOH/DMF to afford compounds **3a-e**, **5a-e**, and **7a-c**, respectively.

##### 3-((4-Acetylbenzylidene)hydrazono)indolin-2-one (3a)

Yield 73%, m.p. 228–230 °C; IR (KBr, *ν* cm^−1^): 3184 (NH), 1722 and 1660 (2 × C=O), 1616 (C=N); ^1^H NMR (DMSO-d_6_) *δ*: 2.64 (s, 3H, CH_3_), 6.91 (d, *J* = 7.5 Hz, 1H, H-7 isatin), 7.03 (t, *J* = 7.5 Hz, 1H, H-5 isatin), 7.42 (t, *J* = 7.5 Hz, 1H, H-6 isatin), 7.80 (d, *J* = 7.5 Hz, 1H, H-4 isatin), 8.04–8.14 (m, 4H, of 4-COCH_3_-C_6_H_4_), 8.64 (s, 1H, –CH=N), 10.91 (s, 1H, NH); ^13^C NMR (DMSO-d_6_) *δ*: 27.4 (CH_3_), 111.5, 116.7, 122.9, 129.1, 129.2, 129.3, 129.4, 134.5, 137.7, 139.4, 145.7, 150.4, 158.3, 164.8 (C=O), 198.1 (C=O); MS *m/z*: 291 [M^+^]; Anal. Calcd for C_17_H_13_N_3_O_2_ (291.30): C, 70.09; H, 4.50; N, 14.42; Found C, 69.88; H, 4.56; N, 14.51.

##### Methyl 4-(((2-oxoindolin-3-ylidene)hydrazono)methyl)benzoate (3b)

Yield 70%, m.p. 225–227 °C; IR (KBr, *ν* cm^−1^): 3275 (NH), 1724 and 1675 (2 × C=O), 1612 (C=N); ^1^H NMR (DMSO-d_6_) *δ*: 3.91 (s, 3H, CH_3_), 6.92 (d, *J* = 8.0 Hz, 1H, H-7 isatin), 7.01 (t, 1H, H-5 isatin, *J* = 8.0 Hz), 7.41 (t, *J* = 8.0 Hz, 1H, H-6 isatin), 7.51 (d, *J* = 7.5 Hz, 1H, H-4 isatin), 8.09–8.13 (m, 4H, of 4-COOCH_3_-C_6_H_4_), 8.65 (s, 1H, –CH=N), 11.00 (s, 1H, NH); ^13^C NMR (DMSO-d_6_) *δ*: 53.1 (COOCH_3_), 116.2, 123.1, 128.8, 129.5, 130.4, 134.9, 137.4, 138.1 (2C), 145.2, 145.5, 150.4, 158.5, 164.10, 164.9, 166.1 (C=O); MS *m/z*: 307 [M^+^]; Anal. Calcd for C_17_H_13_N_3_O_3_ (307.30): C, 66.44; H, 4.26; N, 13.67; Found C, 66.69; H, 4.21; N, 13.52.

##### Methyl 4-(((5-chloro-2-oxoindolin-3-ylidene)hydrazono)methyl)benzoate (3c)

Yield 65%, m.p. 266–268 °C; IR (KBr, *ν* cm^−1^): 3215 (NH), 1734 and 1680 (2 × C=O), 1616 (C=N); ^1^H NMR (DMSO-d_6_) *δ*: 3.91 (s, 3H, CH_3_), 6.94 (d, *J* = 8.0 Hz, 1H, H-7 isatin), 7.49 (d, *J* = 8.0 Hz, 1H, H-6 isatin), 7.74 (s, 1H, H-4 isatin), 8.08–8.15 (m, 4H, of 4-COOCH_3_-C_6_H_4_), 8.68 (s, 1H, –CH=N), 11.06 (s, 1H, NH); ^13^C NMR (DMSO-d_6_) *δ*: 52.9 (COOCH_3_), 113.1, 117.6, 126.4, 128.4, 129.4, 130.5, 132.8, 133.9, 134.4, 137.7, 144.4, 149.8, 159.3, 163.8, 164.5, 166.1 (C=O); MS *m/z*: 341 [M^+^]; Anal. Calcd for C_17_H_12_ClN_3_O_3_ (341.75): C, 59.75; H, 3.54; N, 12.30; Found C, 60.02; H, 3.49; N, 12.18.

##### 3-((4-Acetylbenzylidene)hydrazono)-5-bromoindolin-2-one (3d)

Yield 80%, m.p. 270–272 °C; IR (KBr, *ν* cm^−1^): 3178 (NH), 1751 and 1718 (2 × C=O), 1612 (C=N); ^1^H NMR (DMSO-d_6_) *δ*: 2.65 (s, 3H, CH_3_), 6.88 (d, *J* = 8.0 Hz, 1H, H-7 isatin), 7.59 (d, *J* = 7.5 Hz, 1H, H-6 isatin), 7.89 (s, 1H, H-4 isatin), 8.07 (d, *J* = 8.0 Hz, 2H, H-2 and H-6 of 4-COCH_3_-C_6_H_4_), 8.14 (d, *J* = 8.5 Hz, 2H, H-3 and H-5 of 4-COCH_3_-C_6_H_4_), 8.67 (s, 1H, –CH=N), 11.06 (s, 1H, NH); ^13^C NMR (DMSO-d_6_) *δ*: 27.5 (CH_3_), 113.5, 113.9, 118.3, 129.3, 129.5, 131.2, 136.6, 137.5, 139.5, 144.8, 145.9, 149.1, 159.4, 164.3 (C=O), 198.1 (C=O); MS *m/z*: 370 [M^+^]; Anal. Calcd for C_17_H_12_BrN_3_O_2_ (370.20): C, 55.15; H, 3.27; N, 11.35; Found C, 54.89; H, 3.33; N, 11.49.

##### Methyl 4-(((5-bromo-2-oxoindolin-3-ylidene)hydrazono)methyl)benzoate (3e)

Yield 85%, m.p. 283–285 °C; IR (KBr, *ν* cm^−1^): 3157 (NH), 1705 and 1685 (2 × C=O), 1618 (C=N); ^1^H NMR (DMSO-d_6_) *δ*: 3.91 (s, 3H, CH_3_), 6.89 (d, *J* = 8.5 Hz, 1H, H-7 isatin), 7.60 (dd, *J* = 8.5, 2.0 Hz, 1H, H-6 isatin), 7.86 (d, *J* = 2.0 Hz, 1H, H-4 isatin), 8.08 (d, *J* = 8.5 Hz, 2H, H-2 and H-6 of 4-COOCH_3_-C_6_H_4_), 8.15 (d, *J* = 8.0 Hz, 2H, H-3 and H-5 of 4-COOCH_3_-C_6_H_4_), 8.67 (s, 1H, –CH=N), 11.07 (s, 1H, NH); ^13^C NMR (DMSO-d_6_) *δ*: 52.9 (COOCH_3_), 113.5, 113.9, 118.3, 129.3 (2 × C), 130.4 (2 × C), 131.2, 132.7, 136.6, 137.7, 144.8, 149.6, 159.0, 164.3, 166.1 (C=O); MS *m/z*: 386 [M^+^]; Anal. Calcd for C_17_H_12_BrN_3_O_3_ (386.20): C, 52.87; H, 3.13; N, 10.88; Found, 53.10; H, 3.08; N, 10.72.

##### 3-(((4-Methylthiazol-2-yl)methylene)hydrazono)indolin-2-one (5a)

Yield 70%, m.p. 238–240 °C; IR (KBr, *ν* cm^−1^): 3199 (NH), 1732 (C=O), 1614 (C=N); ^1^H NMR (DMSO-d_6_) *δ*: 2.45 (s, 3H, CH_3_), 6.91 (dd, *J* = 7.5, 3.5 Hz, 1H, H-7 isatin), 7.03 (t, *J* = 7.5 Hz, 1H, H-5 isatin), 7.42 (d × t, *J* = 8.0, 3.5 Hz, 1H, H-6 isatin), 7.68 (s, 1H, H-5 thiazole), 7.75 (d, *J* = 7.5 Hz, 1H, H-4 isatin), 8.69 (s, 1H, –CH=N), 10.93 (s, 1H, NH); ^13^C NMR (DMSO-d_6_) *δ*: 17.2 (CH_3_), 111.6, 116.6, 120.0, 122.9, 129.3, 134.8, 145.9, 151.0, 153.3, 155.4, 162.5, 164.6 (C=O); MS *m/z*: 270 [M^+^]; Anal. Calcd for C_13_H_10_N_4_OS (270.31): C, 57.76; H, 3.73; N, 20.73; Found C, 57.65; H, 3.75; N, 20.68.

##### 5-Chloro-3-((thiazol-2-ylmethylene)hydrazono)indolin-2-one (5b)

Yield 75%, m.p. 293–295 °C; IR (KBr, *ν* cm^−1^): 3217 (NH), 1722 (C=O), 1614 (C=N); ^1^H NMR (DMSO-d_6_) *δ*: 6.93 (d, *J* = 8.5 Hz, 1H, H-7 isatin), 7.49–755 (m, 2H, Ar-H), 7.79 (s, 1H, H-4 isatin), 8.19 (d, *J* = 3.0 Hz, 1H, Ar-H), 8.82 (s, 1H, –CH=N), 11.08 (s, 1H, NH); ^13^C NMR (DMSO-d_6_) *δ*: 113.2, 117.6, 125.9, 126.5, 128.4, 134.1, 144.7, 146.0, 150.9, 154.9, 163.2, 164.4 (C=O); MS *m/z*: 290 [M^+^]; Anal. Calcd for C_12_H_7_ClN_4_OS (290.73): C, 49.57; H, 2.43; N, 19.27; Found C, 49.70; H, 2.48; N, 19.16.

##### 5-Chloro-3-(((4-methylthiazol-2-yl)methylene)hydrazono)indolin-2-one (5c)

Yield 77%, m.p. 290–292 °C; IR (KBr, *ν* cm^−1^): 3230 (NH), 1718 (C=O), 1614 (C=N); ^1^H NMR (DMSO-d_6_) *δ*: 2.45 (s, 3H, CH_3_), 6.90 (d, *J* = 8.0 Hz, 1H, H-7 isatin), 7.61 (d, *J* = 8.0, 2.0 Hz, 1H, H-6 isatin), 7.75 (s, 1H, H-5 thiazole), 7.81 (s, 1H, H-4 isatin), 8.73 (s, 1H, –CH=N), 11.10 (s, 1H, NH); ^13^C NMR (DMSO-d_6_) *δ*: 17.2 (CH_3_), 113.1, 117.6, 120.6, 126.4, 128.4, 128.9, 134.1, 144.7, 146.3, 155.1, 162.2, 164.4 (C=O); MS *m/z*: 304 [M^+^]; Anal. Calcd for C_13_H_9_ClN_4_OS (304.75): C, 51.23; H, 2.98; N, 18.38; Found C, 51.39; H, 3.03; N, 18.23.

##### 5-Bromo-3-((thiazol-2-ylmethylene)hydrazono)indolin-2-one (5d)

Yield 83%, m.p. 260–262 °C; IR (KBr, *ν* cm^−1^): 3236 (NH), 1718 (C=O), 1612 (C=N); ^1^H NMR (DMSO-d_6_) *δ*: 6.89 (d, *J* = 8.0 Hz, 1H, H-7 isatin), 7.61 (d, *J* = 8.5 Hz, 1H, H-6 isatin), 7.93 (s, 1H, H-4 isatin), 8.16 (d, *J* = 3.0 Hz, 1H, H-5 thiazole), 8.20 (d, *J* = 3.0 Hz, 1H, H-4 thiazole), 8.81 (s, 1H, –CH=N), 11.08 (s, 1H, NH); ^13^C NMR (DMSO-d_6_) *δ*: 118.3, 125.9, 130.4, 131.7, 136.9, 145.1, 145.3, 146.1, 150.9, 154.9, 163.2, 164.2 (C=O); MS *m/z*: 335 [M^+^]; Anal. Calcd for C_12_H_7_BrN_4_OS (335.18): C, 43.00; H, 2.11; N, 16.72; Found C, 42.79; H, 2.14; N, 16.85.

##### 5-Bromo-3-(((4-methylthiazol-2-yl)methylene)hydrazono)indolin-2-one (5e)

Yield 80%, m.p. 248–250 °C; IR (KBr, *ν* cm^−1^): 3138 (NH), 1735 (C=O), 1612 (C=N); ^1^H NMR (DMSO-d_6_) *δ*: 2.46, 2.48 (2s, 3H, CH_3_), 6.88 (d, *J* = 8.0 Hz, 1H, H-7 isatin), 7.60 (dd, *J* = 8.0, 2.0 Hz, 1H, H-6 isatin), 7.72, 7.83 (2s, 1H, H-5 thiazole), 7.90, 7.94 (2d, *J* = 2.0 Hz, 1H, H-4 isatin), 8.53, 8.75 (2 × s, 1H, –CH=N), 11.07, 11.11 (2 × s, 1H, NH); ^13^C NMR (DMSO-d_6_) *δ*: 16.9, 17.2 (2 × CH_3_), 113.6, 114.1, 115.7, 118.0, 118.4, 120.6, 121.4, 130.9, 131.7, 132.4, 135.4, 136.8, 136.9, 144.8, 145.0, 150.9, 152.3, 154.9 155.1, 155.6, 162.2, 164.1, 164.2 (C=O); MS *m/z*: 349 [M^+^]; Anal. Calcd for C_13_H_9_BrN_4_OS (349.21): C, 44.71; H, 2.60; N, 16.04; Found C, 44.90; H, 2.54; N, 16.15.

##### 3-(((1H-pyrazol-5-yl)methylene)hydrazono)indolin-2-one (7a)

Yield 74%, m.p. 283–285 °C; IR (KBr, *ν* cm^−1^): 3215 (NH), 1708 (C=O), 1618 (C=N); ^1^H NMR (DMSO-d_6_) *δ*: 6.93–7.05 (m, 3H, Ar-H), 7.39 (t, *J* = 7.5 Hz, 1H, H-6 isatin), 7.51 (d, *J* = 7.5 Hz, 1H, H-4 isatin), 7.96–8.00 (m, 1H, Ar-H), 8.55 (s, 1H, –CH=N), 11.01 (s, 1H, NH), 13.54 (s, 1H, NH); ^13^C NMR (DMSO-d_6_) *δ*: 102.9, 111.2, 118.6, 122.8, 123.0, 131.7, 134.9, 142.0, 145.4, 145.9, 156.3, 165.0 (C=O); MS *m/z*: 239 [M^+^]; Anal. Calcd for C_12_H_9_N_5_O (239.23): C, 60.25; H, 3.79; N, 29.27; Found C, 60.07; H, 3.82; N, 29.39.

##### 3-(((1H-pyrazol-5-yl)methylene)hydrazono)-5-chloroindolin-2-one (7b)

Yield 77%, m.p. >300 °C; IR (KBr, *ν* cm^−1^): 3221 (NH), 1735 (C=O), 1620 (C=N); ^1^H NMR (DMSO-d_6_) *δ*: 6.90 (d, *J* = 9.0 Hz, 1H, Ar-H), 6.94 (d, *J* = 8.0 Hz, 1H, Ar-H), 7.46 (dd, *J* = 8.0, 2.5 Hz, 1H, Ar-H), 7.74 (d, *J* = 2.5 Hz, 1H, Ar-H), 7.96 (d, *J* = 8.0 Hz, 1H, Ar-H), 8.59 (s, 1H, –CH=N), 11.00 (s, 1H, NH), 13.17 (s, 1H, NH); ^13^C NMR (DMSO-d_6_) *δ*: 103.0, 112.5, 118.9, 125.7, 127.5, 135.9, 137.1, 140.5, 144.7, 147.1, 157.1, 164.2 (C=O); MS *m/z*: 273 [M^+^]; Anal. Calcd for C_12_H_8_ClN_5_O (273.68): C, 52.66; H, 2.95; N, 25.59; Found C, 52.43; H, 3.00; N, 25.75.

##### 3-(((1H-pyrazol-5-yl)methylene)hydrazono)-5-bromoindolin-2-one (7c)

Yield 85%, m.p. 264–266 °C; IR (KBr, *ν* cm^−1^): 3209 (NH), 1734 (C=O), 1612 (C=N); ^1^H NMR (DMSO-d_6_) *δ*: 6.82 (d, *J* = 8.5 Hz, 1H, Ar-H), 6.87 (d, *J* = 8.0 Hz, 1H, Ar-H), 7.58 (d, *J* = 8.5 Hz, 1H, Ar-H), 7.94 (s, 1H, Ar-H), 8.10 (d, *J* = 8.0 Hz, 1H, Ar-H), 8.60 (s, 1H, –CH=N), 11.04 (s, 1H, NH), 13.62 (s, 1H, NH); ^13^C NMR (DMSO-d_6_) *δ*: 104.76, 113.28, 118.65, 122.19, 127.95, 131.43, 136.39, 144.51, 147.30, 150.39, 156.64, 164.61 (C=O); MS *m/z*: 318 [M^+^]; Anal. Calcd for C_12_H_7b_rN_5_O (318.13): C, 45.31; H, 2.53; N, 22.01; Found C, 45.49; H, 2.55; N, 21.86.

#### General procedure for the synthesis of thiazoles 10a-l

A mixture of previously reported thiosemicarbazones **8a-c**^23^ (2 mmol) and bromoethanones (**9a-d**) (2 mmol) in absolute ethanol (20 ml) was heated under reflux for 3 h, then left to cool. The precipitated solid was collected by filtration, washed with ethanol, dried and recrystallised from EtOH/DMF to yield the corresponding thiazole derivatives **10a-l**, respectively. Compounds **10a-e** and **10g-l**^24–29^.

##### 3–(2-(4-Phenylthiazol-2-yl)hydrazono)indolin-2-one (10a)

Yield 70%, m.p. 275–277 °C; IR (KBr, *ν* cm^−1^): 3136 (NH), 1707 (C=O), 1616 (C=N); ^1^H NMR (DMSO-d_6_) *δ*:6.96 (dd, *J* = 8.0, 3.5 Hz, 1H, H-7 isatin), 7.09 (t, *J* = 7.5 Hz, 1H, H-5 isatin), 7.30–7.54 (m, 6H, Ar-H), 7.64 (s, 1H, H-5 thiazole), 7.89 (d, *J* = 7.5 Hz, 1H, H-4 isatin), 11.26 (s, 1H, NH), 13.32 (s, 1H, NH); ^13^C NMR (DMSO-d_6_) *δ*: 107.3, 111.6, 119.9, 120.4, 121.8, 122.9, 126.0, 128.5, 129.2, 130.9, 132.6, 134.4, 141.8, 148.8, 151.5, 163.7, 166.5 (C=O); MS *m/z*: 320 [M^+^]; Anal. Calcd for C_17_H_12_N_4_OS (320.37) C, 63.73; H, 3.78; N, 17.49; Found, C, 63.91; H, 3.81; N, 17.49.

##### 3–(2-(4–(4-Tolyl)thiazol-2-yl)hydrazono)indolin-2-one (10b)

Yield 67%, m.p. 284–286 °C; IR (KBr, *ν* cm^−1^): 3315 (NH), 1707 (C=O), 1612 (C=N); ^1^H NMR (DMSO-d_6_) *δ*: 2.38 (s, 3H, CH_3_), 6.96 (dd, *J* = 8.0, 3.5 Hz, 1H, H-7 isatin), 7.08 (t, *J* = 7.5 Hz, 1H, H-5 isatin), 7.20–7.38 (m, 3H, Ar-H), 7.53 (d, *J* = 8.0 Hz, 1H, H-4 isatin), 7.68 (s, 1H, H-5 thiazole), 7.79 (d, *J* = 8.5 Hz, 2H, H-2 and H-6 of 4-CH_3_-C_6_H_4_), 11.26 (s, 1H, NH), 13.33 (s, 1H, NH); ^13^C NMR (DMSO-d_6_) *δ*: 21.3 (CH_3_), 119.9, 120.3, 121.8, 122.9, 126.1, 128.3, 129.5, 129.8, 130.6, 130.9, 131.8, 132.5, 137.8, 141.75, 151.5, 163.5, 166.4 (C=O); MS *m/z*: 334 [M^+^]; Anal. Calcd for C_18_H_14_N_4_OS (334.39): C, 64.65; H, 4.22; N, 16.75; Found, C, 64.43; H, 4.24; N, 16.87.

##### 3–(2-(4–(4-Chlorophenyl)thiazol-2-yl)hydrazono)indolin-2-one (10c)

Yield 73%, m.p. 295–297 °C; IR (KBr, *ν* cm^−1^): 3257 (NH), 1693 (C=O), 1618 (C=N); ^1^H NMR (DMSO-d_6_) *δ*: 6.95 (d, *J* = 7.5 Hz, 1H, H-7 isatin), 7.06 (t, *J* = 7.5 Hz, 1H, H-5 isatin), 7.32 (t, *J* = 7.5 Hz, 1H, H-6 isatin,), 7.45 (d, *J* = 8.5 Hz, 2H, H-3 and H-5 of 4-Cl-C_6_H_4_), 7.51 (d, *J* = 8.0 Hz, 1H, H-4 isatin), 7.67 (s, 1H, H-5 thiazole), 7.90 (d, *J* = 8.5 Hz, 2H, H-2 and H-6 of 4-Cl-C_6_H_4_), 11.24 (s, 1H, NH), 13.33 (s, 1H, NH); ^13^C NMR (DMSO-d_6_) *δ*: 107.9, 111.5, 120.2, 120.3, 122.9, 127.8, 127.9, 128.9, 129.1, 130.1, 130.9, 132.7, 133.3, 141.8, 150.3, 163.6, 166.7 (C=O); MS *m/z*: 354 [M^+^]; Anal. Calcd for C_17_H_11_ClN_4_OS (354.81) C, 57.55; H, 3.12; N, 15.79; Found, C, 57.76; H, 3.17; N, 15.66.

##### 3–(2-(4–(4-Bromophenyl)thiazol-2-yl)hydrazono)indolin-2-one (10d)

Yield 75%, m.p. >300 °C; IR (KBr, *ν* cm^−1^): 3259 (NH), 1689 (C=O), 1618 (C=N); ^1^H NMR (DMSO-d_6_) *δ*: 6.95 (d, *J* = 7.5 Hz, 1H, H-7 isatin), 7.09 (t, *J* = 7.5 Hz, 1H, H-5 isatin), 7.33 (t, *J* = 7.5 Hz, 1H, H-6 isatin), 7.52 (d, *J* = 7.5 Hz, 1H, H-4 isatin), 7.60 (d, *J* = 8.0 Hz, 2H, H-3 and H-5 of 4-Br-C_6_H_4_), 7.68 (s, 1H, H-5 thiazole), 7.84 (d, *J* = 8.5 Hz, 2H, H-2 and H-6 of 4-Br-C_6_H_4_), 11.25 (s, 1H, NH), 13.34 (s, 1H, NH); ^13^C NMR (DMSO-d_6_) *δ*: 108.1, 111.5, 120.2, 120.4, 121.5, 122.9, 128.0, 128.2, 131.0, 131.9, 132.1, 132.7, 133.6, 141.8, 150.3, 163.7, 166.2 (C=O); MS *m/z*: 399 [M^+^]; Anal. Calcd for C_17_H_11_BrN_4_OS (399.26) C, 51.14; H, 2.78; N, 14.03; Found, C, 50.89; H, 2.81; N, 14.14.

##### 5-Chloro-3–(2-(4-phenylthiazol-2-yl)hydrazono)indolin-2-one (10e)

Yield 80%, m.p. 292–294 °C; IR (KBr, *ν* cm^−1^): 3169 (NH), 1707 (C=O), 1612 (C=N); ^1^H NMR (DMSO-d_6_) *δ*:6.95 (d, *J* = 7.5 Hz, 1H, H-7 isatin), 7.30–7.60 (m, 6H, Ar-H), 7.68 (s, 1H, H-5 thiazole), 7.84 (d, *J* = 7.5 Hz, 1H, H-4 isatin), 11.25 (s, 1H, NH), 13.34 (s, 1H, NH); ^13^C NMR (DMSO-d_6_) *δ*: 108.1, 111.5, 120.2, 120.4, 121.5, 122.9, 128.19, 131.0, 132.1, 132.7, 133.6, 141.8, 150.3, 163.7, 166.7 (C=O); MS *m/z*: 354 [M^+^]; Anal. Calcd for C_17_H_11_ClN_4_OS (354.81) C, 57.55; H, 3.12; N, 15.79; Found, C, 57.73; H, 3.07; N, 15.64.

##### 5-Chloro-3–(2-(4-(p-tolyl)thiazol-2-yl)hydrazono)indolin-2-one (10f)

Yield 78%, m.p. >300 °C; IR (KBr, *ν* cm^−1^): 3155 (NH), 1685 (C=O), 1618 (C=N); ^1^H NMR (DMSO-d_6_) *δ*: 2.38, 2.40 (2s, 3H, CH_3_), 6.85, 6.97 (2 × d, *J* = 8.5 Hz, 1H, H-7 isatin), 7.28–7.38 (m, 3H, Ar-H), 7.52, 7.58 (2s, 1H, H-5 thiazole), 7.71, 7.78 (2 × d, *J* = 8.5 Hz, 2H, H-2 and H-6 of 4-CH_3_-C_6_H_4_), 8.34, 8.41 (2 × d, *J* = 2.0 Hz, 1H, H-4 isatin), 10.62, 11.36 (2s, 1H, NH), 13.34 (s, br, 1H, NH); ^13^C NMR (DMSO-d_6_) *δ*: 21.3 (CH_3_), 106.9, 111.5, 113.0, 119.8, 121.9, 126.0, 126.1, 127.0, 129.8, 129.9, 131.3, 131.7, 137.9, 140.3, 151.6, 163.5, 166.2 (C=O); MS *m/z*: 368 [M^+^]; Anal. Calcd for C_18_H_13_ClN_4_OS (368.84) C, 58.61; H, 3.55; N, 15.19; Found, C, 58.77; H, 3.51; N, 15.07.

##### 5-Chloro-3–(2-(4–(4-chlorophenyl)thiazol-2-yl)hydrazono)indolin-2-one (10g)

Yield 85%, m.p. >300 °C; IR (KBr, *ν* cm^−1^): 3215 (NH), 1695 (C=O), 1620 (C=N); ^1^H NMR (DMSO-d_6_) *δ*: 6.98 (d, 1H, H-7 isatin, *J* = 8.5 Hz), 7.37 (dd, *J* = 8.5, 2.0 Hz, 1H, H-6 isatin), 7.48 (d, *J* = 8.5 Hz, 2H, H-3 and H-5 of 4-Cl-C_6_H_4_), 7.53 (d, *J* = 2.0 Hz, 1H, H-4 isatin), 7.74 (s, 1H, H-5 thiazole), 7.92 (d, *J* = 8.5 Hz, 2H, H-2 and H-6 of 4-Cl-C_6_H_4_), 11.37 (s, 1H, NH), 13.34 (s, 1H, NH); ^13^C NMR (DMSO-d_6_) *δ*: 108.5, 113.1, 119.8, 121.9, 127.1, 127.8, 127.9, 129.2, 129.5, 130.3, 131.6, 132.3, 133.2, 140.4, 150.3, 163.5, 166.5 (C=O); MS *m/z*: 389 [M^+^]; Anal. Calcd for C_17_H_10_Cl_2_N_4_OS (389.26): C, 52.45; H, 2.59; N, 14.39; Found, C, 52.67; H, 2.62; N, 14.53.

##### 3–(2-(4–(4-Bromophenyl)thiazol-2-yl)hydrazono)-5-chloroindolin-2-one (10h)

Yield 90%, m.p. >300 °C; IR (KBr, *ν* cm^−1^): 3250 (NH), 1690 (C=O), 1620 (C=N); ^1^H NMR (DMSO-d_6_) *δ*: 6.97 (d, *J* = 8.0 Hz, 1H, H-7 isatin), 7.37 (dd, *J* = 8.5, 2.0 Hz, 1H, H-6 isatin), 7.53 (d, *J* = 2.0 Hz, 1H, H-4 isatin), 7.61 (d, *J* = 8.5 Hz, 2H, H-3 and H-5 of 4-Br-C_6_H_4_), 7.75 (s, 1H, H-5 thiazole), 7.85 (d, *J* = 8.5 Hz, 2H, H-2 and H-6 of 4-Br-C_6_H_4_), 11.37 (s, 1H, NH), 13.34 (s, 1H, NH); ^13^C NMR (DMSO-d_6_) *δ*: 108.5, 113.1, 120.0, 121.9, 122.0, 126.7, 128.1, 128.3, 130.6, 131.2, 132.1, 132.3, 133.6, 140.4, 150.4, 163.5, 166.7 (C=O); MS *m/z*: 433 [M^+^]; Anal. Calcd for C_17_H_10_BrClN_4_OS (433.71) C, 47.08; H, 2.32; N, 12.92; Found, C, 46.87; H, 2.29; N, 13.11.

##### 5-Bromo-3–(2-(4-phenylthiazol-2-yl)hydrazono)indolin-2-one (10i)

Yield 83%, m.p. 285–287 °C; IR (KBr, *ν* cm^−1^): 3169 (NH), 1685 (C=O), 1614 (C=N); ^1^H NMR (DMSO-d_6_) *δ*:6.91 (d, *J* = 8.0 Hz, 1H, H-7 isatin), 7.42–7.84 (m, 6H, Ar-H), 7.90 (d, *J* = 8.0 Hz, 1H, Ar-H), 8.53 (d, *J* = 8.0 Hz, 1H, H-4 isatin), 11.37 (s, 1H, NH), 13.32 (s, 1H, NH); ^13^C NMR (DMSO-d_6_) *δ*: 107.8, 112.0, 113.5, 114.7, 122.4, 122.5, 126.1, 128.5, 129.2, 129.4, 131.3, 133.1, 134.4, 140.7, 151.6, 163.3, 166.3 (C=O); MS *m/z*: 399 [M^+^]; Anal. Calcd for C_17_H_11_BrN_4_OS (399.26) C, 51.14; H, 2.78; N, 14.03; Found, C, 51.37; H, 2.72; N, 13.88.

##### 5-Bromo-3–(2-(4–(4-tolyl)thiazol-2-yl)hydrazono)indolin-2-one (10j)

Yield 80%, m.p. >300 °C; IR (KBr, *ν* cm^−1^): 3157 (NH), 1685 (C=O), 1618 (C=N); ^1^H NMR (DMSO-d_6_) *δ*: 2.38, 2.39 (2s, 3H, CH_3_), 6.80, 6.92 (2 × d, *J* = 8.0 Hz, 1H, H-7 isatin), 7.18, 7.24 (2 × d, *J* = 7.5 Hz, 2H, H-3 and H-5 of 4-CH_3_-C_6_H_4_), 7.41, 7.49 (2 × dd, *J* = 8.0, 2.0 Hz, 1H, H-6 isatin), 7.59 (s, 1H, H-5 thiazole), 7.63, 8.52 (2 × d, *J* = 2.0 Hz, 1H, H-4 isatin), 7.72, 7.78 (2 × d, *J* = 8.0 Hz, 2H, H-2 and H-6 of 4-CH_3_-C_6_H_4_), 10.62, 11.37 (2s, 1H, NH), 12.97, 13.32 (s, 1H, NH); ^13^C NMR (DMSO-d_6_) *δ*: 21.3 (CH_3_), 106.9, 113.5, 114.7, 122.4, 122.5, 126.0, 126.1, 129.8, 129.9, 131.2, 131.7, 133.0, 137.9, 140.7, 151.7, 163.3, 166.2 (C=O); MS *m/z*: 413 [M^+^]; Anal. Calcd for C_18_H_13_BrN_4_OS (413.29) C, 52.31; H, 3.17; N, 13.56; Found, C, 52.59; H, 3.14; N, 13.48.

##### 5-Bromo-3–(2-(4–(4-chlorophenyl)thiazol-2-yl)hydrazono)indolin-2-one (10k)

Yield 84%, m.p. >300 °C; IR (KBr, *ν* cm^−1^): 3159 (NH), 1687 (C=O), 1614 (C=N); ^1^H NMR (DMSO-d_6_) *δ*: 6.92 (d, *J* = 8.5 Hz, 1H, H-7 isatin), 7.48–7.57 (m, 4H, Ar-H), 7.64 (s, 1H, H-5 thiazole), 7.92 (d, *J* = 8.5 Hz, 2H, H-2 and H-6 of 4-Cl-C_6_H_4_), 11.37 (s, 1H, NH), 13.32 (s, 1H, NH); ^13^C NMR (DMSO-d_6_) *δ*: 108.8, 113.4, 115.1, 122.2, 122.5, 127.9 (2 × C), 129.2, 129.8, 131.5, 132.9, 133.1, 133.2, 140.9, 150.3, 163.4, 166.5 (C=O); MS *m/z*: 433 [M^+^]; Anal. Calcd for C_17_H_10_BrClN_4_OS (433.71) C, 47.08; H, 2.32; N, 12.92; Found, C, 47.15; H, 2.35; N, 12.81.

##### 5-Bromo-3–(2-(4–(4-bromophenyl)thiazol-2-yl)hydrazono)indolin-2-one (10l)

Yield 90%, m.p. >300 °C; IR (KBr, *ν* cm^−1^): 3157 (NH), 1685 (C=O), 1614 (C=N); ^1^H NMR (DMSO-d_6_) *δ*: 6.93 (d, *J* = 8.5 Hz, 1H, H-7 isatin), 7.50–7.87 (m, 6H, Ar-H), 8.52 (s, 1H, H-4 isatin), 11.38 (s, 1H, NH), 13.33 (s, 1H, NH); ^13^C NMR (DMSO-d_6_) *δ*: 108.7, 113.6, 114.6, 121.7, 122.3, 122.8, 128.1, 128.2, 131.4, 132.1, 133.1, 133.6, 140.9, 142.1, 150.3, 163.4, 166.5 (C=O); MS *m/z*: 478 [M^+^]; Anal. Calcd for C_17_H_10_Br_2_N_4_OS (478.16) C, 42.70; H, 2.11; N, 11.72; Found, C, 42.52; H, 2.09; N, 11.78.

#### Biological evaluation

##### In vitro anti-proliferative activity

The anti-proliferative activity of the title compounds **3a-e**, **5a-e**, **7a-c**, and **10a-l** towards cancer cell lines, normal cell lines, and multidrug-resistant lung cancer NCI-H69AR cell line was determined using CellTiter-Glo™ Luminescent cell viability assay according to the previously published procedures^14^^,^^15^.

##### Cell cycle effects

Cell cycle effects of the synthesised compounds **7b** and **10e** were determined adopting the reported procedures^14^^,^^15^.

## Results and discussion

### Chemistry

**Scheme 1.**
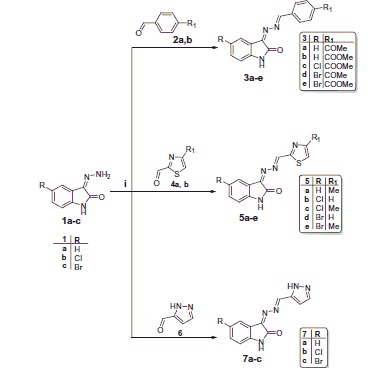
Synthesis of the hydrazonoindolin-2-ones **3a-e**, **5a-e**, and **7a-c**. Reagents and conditions: (**i**) Methanol, glacial acetic acid (cat.), reflux, 4 h.

**Scheme 2.**
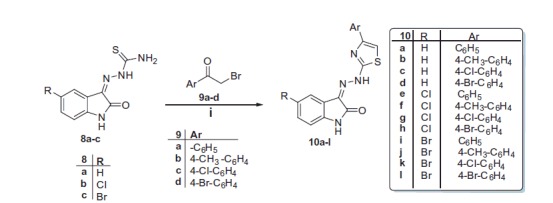
Synthesis of thiazoles **10a-l**. Reagents and conditions: (**i**) Ethanol, reflux, 3 h.

Our proposed synthetic strategy to the target hydrazonoindolin-2-ones **3a-e**, **5a-e**, **7a-c**, and **10a-l** is depicted in Schemes 1 and 2. The synthesis of the 3-hydrazonoindolin-2-ones **1a-c** was accomplished *via* refluxing indoline-2,3-diones with hydrazine hydrate in ethanol. Further condensation of hydrazones **1a-c** with benzaldehydes (**2a**, **b**), thiazole-2-carbaldehydes (**4a**, **b**), or 1*H*-pyrazole-5-carbaldehyde (**6**) in methyl alcohol in the presence of a catalytic amount of glacial acetic acid afforded the target derivatives **3a-e**, **5a-e**, and **7a-c**, respectively, in 65–85% yield (Scheme 1).

The assigned structures of compounds **3a-e**, **5a-e**, and **7a-c** were confirmed by IR, ^1^H and ^13^C NMR spectra. Their IR spectra showed absorption bands around 3200 cm^−1^ for the (NH) groups of the indolin-2-one moiety, in addition to the absorption bands of (C=O) groups in the region of 1660–1751 cm^−1^. ^1^H NMR spectra of compounds **3a-e**, **5a-e**, and **7a-c** revealed the presence of a singlet signal in the region *δ* 10.93–11.11 corresponding to NH proton of isatin moiety, in addition to the singlet signal of the methine (–CH=N–) proton in the region *δ* 8.55–8.82. Also, compounds **7a-c** displayed additional signal attributable to the NH proton of the pyrazole ring at *δ* 13.17–13.62. Moreover, the ^13^C NMR spectra of these compounds displayed signals resonating around *δ* 164 corresponding to the amidic (C=O) carbons.

On the other hand, the condensation of indoline-2,3-diones with thiosemicarbazide in refluxing ethanol containing a catalytic amount of acetic acid yielded the corresponding thiosemicarbazones **8a-c**. The second targeted isatin-based derivatives **10a-l** were obtained in good yields (67–90%) through the reaction of the key intermediates **8a-c** with the appropriate phenacyl bromide **9a-d** in refluxing absolute ethyl alcohol (Scheme 2).

IR spectra of the compounds **10a-l** displayed the characteristic absorption bands in the regions 3136–3315 cm^−1^ and 1685–1707 cm^−1^ corresponding to the (NH) and (C=O) groups, respectively, while their ^1^H NMR spectra were characterised by the presence of three singlets in the regions *δ* 11.24–11.38, 13.32–13.36, and 7.58–7.75 which were assigned to two NH groups and H-5 of the thiazole ring, respectively. Besides, ^13^C NMR spectra of these compounds showed characteristic signals resonating around *δ* 166 which was attributed to (C=O) carbon. It is worth noting that some derivatives are existing in *E/Z* geometrical isomerism in the DMSO-solution state, such as compounds **5e**, **10f**, and **10j**^17^.

### Biological evaluation

#### In vitro anti-proliferative activity

The cancer cell growth inhibitory activity of the synthesised 25 compounds **3a-e**, **5a-e**, **7a-c**, and **10a-l** was evaluated *in vitro* towards three human cancer cell lines, namely lung cancer A-549, human colon cancer HT-29, and breast cancer ZR-75 cell lines using CellTiter-Glo™ Luminescent cell viability assay^30^. Sunitinib was included in this assay as a reference drug.

The prepared hydrazonoindolin-2-ones were first evaluated at preliminary anti-proliferative assay against the three cancer cell lines in quadruplicate at a single concentration of 30 µM. The data presented as percentage growth inhibition (GI%) caused by the test compounds (Table 1).

**Table 1.** Anti-proliferative (cell growth inhibitory activity at 30 µM concentration) activity of the target compounds **3a-e, 5a-e, 7a-c**, **10a-l**, and Sunitinib towards HT-29, ZR-75, and A-549 cell lines.

	Growth inhibition %			
Compound No.	A-549	ZR-75	HT-29	Average growth inhibition %
**3a**	12.7 ± 7.1	29.6 ± 24.4	53.6 ± 12.2	32.0
**3b**	68.1 ± 2.8	80.6 ± 7.0	92.3 ± 3.7	80.3
**3c**	93.5 ± 2.8	98.9 ± 2.1	95.9 ± 4.7	96.1
**3d**	17.3 ± 10.2	53.9 ± 14.4	56.8 ± 15.1	42.7
**3e**	−4.2 ± 9.3	55.9 ± 18.2	26.3 ± 24.4	41.1
**5a**	2.4 ± 5.0	40.8 ± 25.7	37.9 ± 26.3	27.0
**5b**	93.6 ± 5.4	95.0 ± 8.2	96.2 ± 4.5	94.9
**5c**	98.8 ± 1.5	98.0 ± 2.6	96.4 ± 4.8	97.7
**5d**	94.8 ± 2.5	96.4 ± 1.5	98.5 ± 3.1	96.6
**5e**	95.3 ± 3.1	96.9 ± 2.4	99.0 ± 1.1	97.1
**7a**	8.2 ± 15.1	60.1 ± 5.8	60.8 ± 13.1	43.0
**7b**	91.5 ± 1.3	82.3 ± 15.2	94.9 ± 1.8	89.6
**7c**	32.8 ± 8.9	19.2 ± 22.8	28.8 ± 17.3	26.9
**10a**	70.7 ± 5.7	76.9 ± 7.1	91.2 ± 8.3	79.6
**10b**	24.4 ± 14.1	62.1 ± 10.7	33.9 ± 21.2	40.1
**10c**	31.4 ± 9.1	77.1 ± 10.9	85.4 ± 7.0	64.6
**10d**	12.3 ± 11.3	53.8 ± 19.2	20.6 ± 26.9	28.9
**10e**	80.4 ± 6.0	89.3 ± 7.2	85.7 ± 10.2	85.1
**10f**	27.4 ± 5.8	50.3 ± 33.6	54.4 ± 19.5	44.0
**10g**	18.9 ± 3.2	52.8 ± 9.4	34.4 ± 23.6	35.4
**10h**	−2.6 ± 7.5	9.0 ± 19.2	−8.3 ± 43.8	9.0
**10i**	92.3 ± 6.2	97.5 ± 3.1	96.3 ± 5.9	95.4
**10j**	85.9 ± 9.1	73.1 ± 3.5	90.1 ± 5.3	83.0
**10k**	72.4 ± 3.5	50.6 ± 11.5	66.8 ± 19.1	63.3
**10l**	−20.1 ± 5.9	40.9 ± 16.7	27.8 ± 4.3	34.4
Sunitinib	59.5 ± 2.3	90.7 ± 4.5	85.7 ± 2.7	78.7

The tested hydrazonoindolin-2-ones displayed different levels of anti-proliferative activity and possessed a distinctive pattern of selectivity against the tested cancer cell lines with average growth inhibition in the range of 9.0–97.7%. Close examination of the GI% values in Table 1, revealed that compounds **3b**, **3c**, **5b-e**, **7b**, **10e**, **10i**, and **10j** are the most active analogs in this study, showing broad spectrum activity toward the tested cell lines with average GI% values >80%. On the other hand, compounds **3a**, **5a**, **7c**, **10d**, **10h**, and **10l** displayed modest growth inhibitory activity (GI% values <35%). It is noteworthy to mention that compounds **3e** and **10l** stimulated the growth of the A-549 cell line and compound **10h** stimulated the growth of both A-549 and HT-29 cell lines.

The quantitative inhibitory concentration 50% (IC_50_) values were evaluated for the active members that displayed effective growth inhibition toward the three cancer cell lines (GI% values >35%) in the preliminary single high dose (30 µM) screening (Table 2). From the displayed results in Table 2, it was obvious that several of the synthesised hydrazonoindolin-2-ones possessed excellent to moderate growth inhibitory activity against the tested cancer cell lines.

**Table 2.** Inhibitory concentration 50% (IC_50_) of anti-proliferative activity of the tested compounds **3b-e, 5b-e, 7a, 7b**, **10a-k**, and Sunitinib toward HT-29, ZR-75, and A-549 cell lines.

	IC_50_ (µM)^a^			
Compound No.	A-549	ZR-75	HT-29	Average IC_50_ (µM)
**3b**	11.25 ± 1.45	9.72 ± 2.52	21.05 ± 1.86	14.01
**4c**	6.07 ± 0.90	4.20 ± 0.57	8.49 ± 1.01	6.25
**4d**	26.09 ± 4.47	NA^b^	NA^b^	>28.69
**3e**	NA^b^	23.33 ± 0.0	NA^b^	>27.77
**5b**	4.87 ± 0.49	2.09 ± 0.12	6.15 ± 0.51	4.37
**5c**	2.15 ± 0.23	1.16 ± 0.39	4.29 ± 0.25	2.53
**5d**	12.53 ± 1.73	12.68 ± 2.40	15.32 ± 1.93	13.51
**5e**	3.54 ± 0.42	2.93 ± 0.46	8.51	7.49
**7a**	22.94 ± 3.51	NA^b^	NA^b^	>27.65
**7b**	2.29 ± 0.21	1.37 ± 0.23	2.75 ± 0.31	2.14
**10a**	9.41 ± 1.29	4.35	19.44 ± 1.03	11.07
**10b**	NA^b^	24.59	NA^b^	>28.19
**10c**	17.2 ± 2.56	4.89	NA^b^	>17.36
**10e**	4.79 ± 0.45	1.95 ± 1.03	7.25 ± 0.83	4.66
**10f**	22.72 ± 3.71	23.41	NA^b^	>25.38
**10g**	6.07 ± 7.41	1.87 ± 0.26	NA^b^	>12.65
**10i**	6.13 ± 0.76	1.96 ± 0.85	8.26 ± 0.86	5.45
**10j**	12.84 ± 1.40	10.91 ± 2.81	15.22 ± 1.10	12.99
**10k**	20.13 ± 3.81	3.61	19.75 ± 1.63	14.49
Sunitinib	10.14 ± 0.8	8.31 ± 2.4	5.87 ± 0.3	8.11

^a^IC_50_ values are the mean ± SD of four separate experiments.

^b^NA: Compounds having IC_50_ value >30 μM.

Investigations of the anti-proliferative activity against A-549 indicated that compounds **5b**, **5c**, **5e**, **7b**, and **10e** (IC_50_ = 4.87 ± 0.49, 2.15 ± 0.23, 3.54 ± 0.42, 2.29 ± 0.21, and 4.79 ± 0.45 µM, respectively) were found to be the most potent counterparts as they were 2, 4.7, 2.8, 4.4 and 2.1 times, respectively, more active than Sunitinib (IC_50_ = 10.14 ± 0.8 µM). Besides, compounds **3c**, **10a**, **10g**, and **10i** possessed excellent anti-proliferative activity against A-549 cells (IC_50_ = 6.07 ± 0.90, 9.41 ± 1.29, 6.07 ± 7.41, and 6.13 ± 0.76 µM, respectively) which are better than the used reference drug Sunitinib. On the other hand, compounds **3e** and **10b** showed weak activity with IC_50_ >30 µM. Concerning activity against ZR-75 cells, compounds **3c**, **5b**, **5c**, **5e**, **7b**, **10a**, **10c**, **10e**, **10g**, **10i**, and **10k** displayed excellent anti-proliferative activity superior to that of Sunitinib with IC_50_ range 1.16–4.89 µM. Whereas, compounds **3d** and **7a** possessed weak activity with IC_50_ >30 µM. Moreover, anti-proliferative evaluation in HT-29 cell line revealed that two members, **5c** and **7b**, only showed superior activity to Sunitinib (IC_50_ = 4.29 ± 0.25 and 2.75 ± 0.31 µM, respectively). Also, compounds **3c**, **5b**, **5e**, **10e**, and **10i** were moderately active with IC_50_ range of 6.15–8.49 µM.

Compound **7b** was the most active congener in the synthesised Schiff bases **3a-e**, **5a-e**, and **7a-c** with an average IC_50_ value of 2.14 µM toward the tested human cancer cell lines being nearly four-fold more potent than the positive control, Sunitinib (average IC_50_ value =8.11 µM). Whereas, compound **10e** was the most active candidate in the arylthizole derivatives **10a-l** with an apparent average IC_50_ value of 4.66 µM against the tested cell lines.

#### Structure activity relationship

Observing the results, we could deduce valuable data about the structure activity relationship (SAR). Firstly, investigation of the impact of the substitution on the pendant phenyl group in compounds **3a-e** revealed that grafting an ester group (compound **3b**; average IC_50 _= 14.01 µM) is more beneficial for activity than acetyl group (compound **3a**; average IC_50 _> 30 µM). The effect of bioisosteric replacement of the pendant phenyl ring in compounds **3a-e** with 2-thiazolyl ring, compounds **5a-e**, or 5-pyrazolyl ring, compounds **7a-c** was explored. Both 2-thiazolyl moiety (compound **5c**; average IC_50_ = 2.53 µM) and 5-pyrazolyl moiety (compound **7b**; average IC_50 _= 2.14 µM) resulted in remarkable increase in the activity in comparison to compounds **3a-e** (compound **3c**; average IC_50 _= 6.25 µM). Also, substitution of the 2-thiazolyl ring in compounds **5a-e** with 4-methyl group resulted in an increase of the anti-proliferative activity (compound **5b**; average IC_50_ = 4.37 µM vs. compound **5c**; average IC_50_ = 2.53 µM).

Regarding phenylthiazolyl series, compounds **10a-l**, the impact of the substitution on the 4-phenyl group was examined. The decreased average IC_50_ values of compounds **10a**, **10e**, and **10i** (11.07, 4.66 and 5.45 µM, respectively) which incorporated an unsubstituted phenyl moiety than that of their corresponding members **10b**, **10f**, and **10j** (>28.19, >25.38, and 12.99 µM, respectively), with 4-methyl substituted phenyl moiety, and their corresponding members **10c**, **10g**, and **10k** (>17.36, >12.65, and 14.49 µM, respectively), with 4-chloro substituted phenyl moiety, indicated that unsubstitution of the phenyl moiety is advantageous. On the other hand, substitution with 4-bromo substituent (compounds **10d**, **10h**, and **10l**) diminished the activity. Finally, we explored the effect of the substitution of the indolin-2-one moiety. Interestingly, grafting 5-chloro substituent was found to be more beneficial for the activity rather than unsubstituted or 5-bromo substituted indolin-2-one moieties throughout the study.

In conclusion, we can deduce that replacing the phenyl moiety of compounds **3a-e** with 2-thiazolyl ring, 5-pyrazolyl ring, or its substitution with alipophilic ester group greatly enhances the anti-proliferative activity. In addition, hybridisation of the indolin-2-one moiety with 4-phenylthiazolylmoiety, compounds **10a-l** seems to be a prosperous approach.

#### Cell cycle effects

The cell cycle consists of a number of complex biochemical pathways and controlled by numerous mechanisms to ensure correct cell division. An important role is played by cyclin/cyclin-dependent kinase (CDK) complexes, which are critical regulators of cell cycle progression and RNA transcription. Deregulation of the cell cycle underlies the aberrant cell proliferation which characterises loss of cell cycle checkpoint control and cancer^31^^,^^32^. Retinoblastoma protein (pRb), a cell cycle regulatory molecule, plays a critical role in control of the G1-to-S phase checkpoint of the cell cycle. pRb suppresses the activity of E2F transcription factors thereby inhibiting transcription of cell cycle promoting genes, in its hypophosphorylated state. Upon phosphorylation, mainly *via* CDKs, phosphorylated pRb dissociates from E2F and permits cell cycle progression^33^^,^^34^. In the current medical era, therapeutic strategies directed against critical cell cycle regulators have stood out as an attractive approach for discovering therapies for many human malignancies.

**Figure 3.**
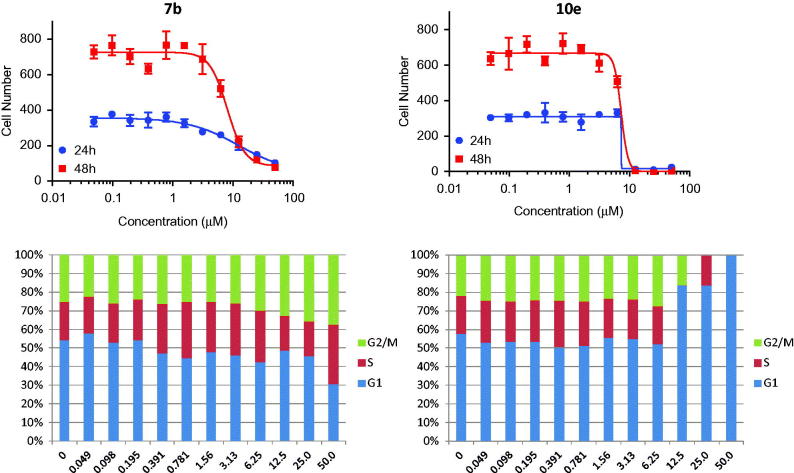
Effects on number and cell cycle distribution. A-549 cells were treated with dose curve. Compound **7b** (left) or **10e** (right). Cell number per cell cycle was determined by DNA content by DAPI fluorescent labeling and imaging cytometry following 48 h treatment.

**Figure 4.**
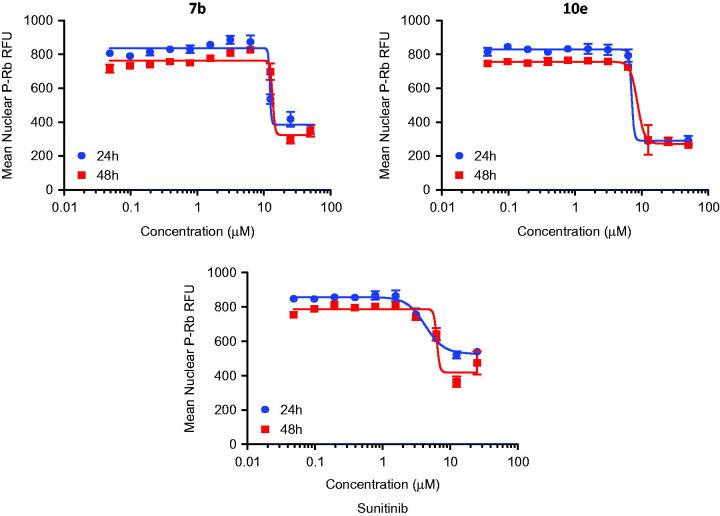
Dose-dependent changes in total levels of phosphorylated Rb protein of **7b**, **10e** and Sunitinib.

**Figure 5.**
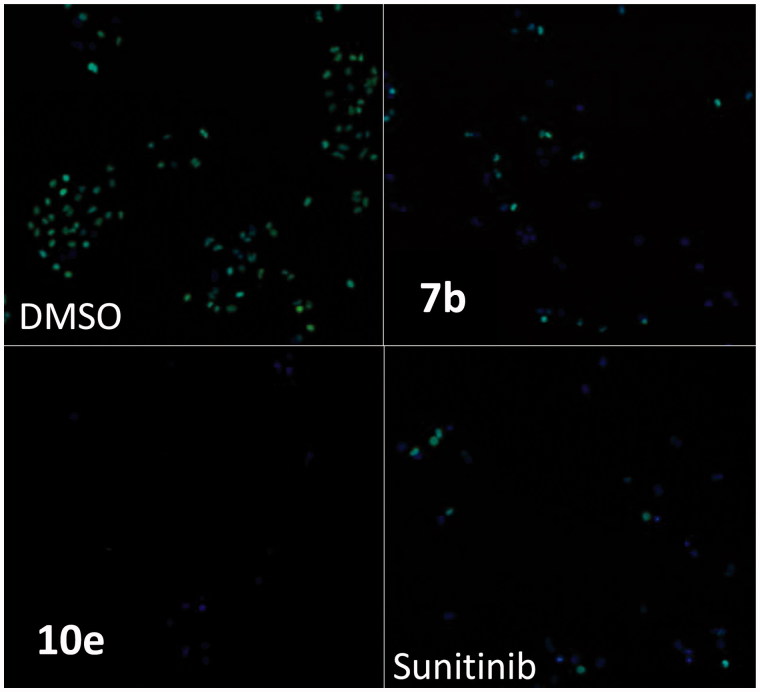
Immunofluorescent imaging of phosphorylated Rb protein for **7b, 10e**, and Sunitinib.

In this study, cancer cells derived from lung adenocarcinoma (A-549) were used to evaluate the effects of compounds **7b** and **10e** on various aspects of the cell cycle progression. Both immunofluorescent imaging of phosphorylated Rb protein and total DNA content of each cell were explored to determine the activity of compounds **7b** and **10e** over a range of concentrations less than 100 nM to 50 µM (Figures 3–5).

Compound **7b** caused a dose-dependent reduction in the total cell number after 48 h of treatment with IC_50_ value =8.08 µM, while compound **10e** caused a dose-dependent reduction in the total cell number after 24 h of treatment with IC_50_ value =7.38 µM (Table 3).

**Table 3.** IC_50_ for reductions in the total cell number and cell cycle effects of compounds **7b**, **10e**, and Sunitinib.

	IC_50_ (µM) for reductions in the total cell number		IC_50_ (µM) for reduction in Rb phosphorylation		
Compound No.	24 h	48 h	24 h	48 h	Cell cycle effects
**7b**	12.67 ± 13.6	8.08 ± 0.95	12.07 ± 0.6	13.56 ± 0.6	G1 decreased and G2/M-phases increased
**10e**	7.38 ± 1.04	7.27 ± 1.15	7.59 ± 0.38	12.46 ± 2.1	G1 increased and G2/M-phases decreased
Sunitinib	12.54 ± 9.82	3.48 ± 0.61	3.18 ± 0.07	14.01 ± 0.6	G1 decreased and G2/M-phases increased

Moreover, compound **7b** caused a decrease in the percentage of cells in the G1 phase of the cell cycle with corresponding increase in the G2/M-phases. Arrest in G2 may represent a checkpoint blockade whereas mitotic arrest may, in some cases, lead to mitotic catastrophe and subsequent programed death of cells with multiple or aberrant nuclei. In contrast, compound **10e** caused an increase in the percentage of cells in the G1 phase of the cell cycle with corresponding decreases in S- and G2/M phases. This suggests that part of the compound effects on growth may be attributable to a decreased rate of progression through the cell cycle and corresponding decreases in proliferation.

However, levels of phosphorylated Rb protein were significantly reduced in a dose-dependent manner by the control and the test compounds **7b** and **10e** (Figure 4). The IC_50_ values were consistent with the IC_50_ values for reductions in cell number caused by each compound after 24 h of treatment while the correlation is less apparent after 48 h of treatment (Table 3). This may support the hypothesis that inhibition of cyclin-dependent kinases by isatin compounds plays a vital role in their growth inhibitory activity.

#### Selectivity

The cell growth inhibitory activity of compounds **7b** and **10e** was examined towards three non-tumorigenic cell lines to estimate their selectivity for tumor cells. A-549 human non-small cell lung cancer (NSCLC) cell line was included in the assay for comparison. Cultures derived from human fibrocystic mammary tissue (MCF-10A) are non-tumorigenic and exhibit features of primary cultures of breast tissue including dome formation^35^. IEC-6 cells derived from rat intestine show morphologic and karyotypic features of normal intestinal epithelial cells^36^. Fibroblasts derived from embryonic tissue from mice (Swiss-3t3) are both non-tumorigenic and contact inhibited^37^. Compounds **7b** and **10e** were tested quadruplicate at maximum concentrations of 25 µM, with subsequent 10 serially diluted concentrations.

**Figure 6.**
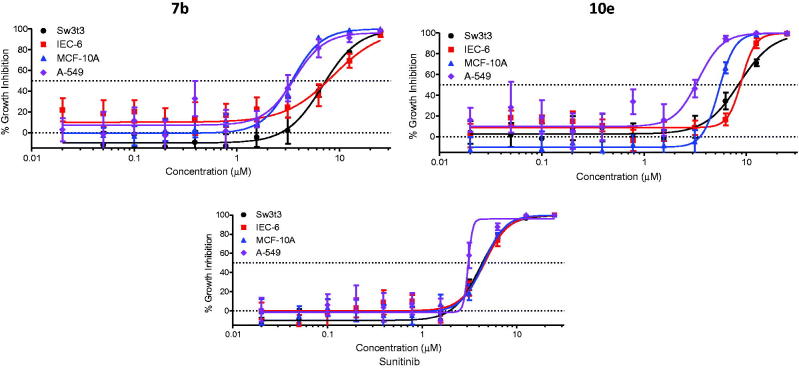
Selectivity profile of **7b, 10e**, and Sunitinib.

The results were displayed in Figure 6, while the IC_50_ values and mean selectivity index were listed in Table 4. Both compounds **7b** and **10e** displayed mean tumor selectivity index (1.6 and 1.8, respectively) higher than that of Sunitinib (1.4).

**Table 4.** Selectivity for compounds **7b**, **10e**, and Sunitinib towards tumor and non-tumorigenic cell lines.

	IC_50_ (µM) ± SD				
Compound No.	Intestine IEC-6	Breast MCF-10A	Fibroblast Swiss-3t3	NSCLC A-549	Mean tumor selectivity
**7b**	8.70 ± 4.32	3.42 ± 0.45	6.85 ± 0.76	3.84 ± 0.67	1.6
**10e**	8.98 ± 1.25	5.37 ± 0.61	8.51 ± 1.01	4.13 ± 0.61	1.8
Sunitinib	4.56 ± 0.54	4.43 ± 0.23	4.07 ± 0.75	3.06 ± 0.9	1.4

#### Multidrug resistant lung cancer cell line (NCI-H69AR)

**Figure 7.**
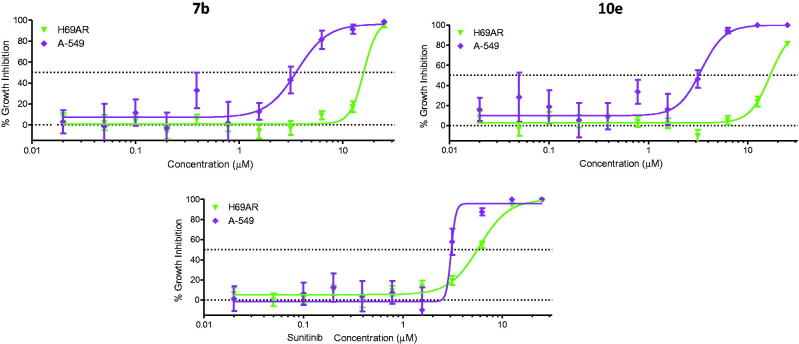
Activity of **7b**, **10e**, and Sunitinib towards sensitive and resistant cancer cell lines.

The anti-proliferative activity of compounds **7b** and **10e** was evaluated towards multidrug resistant lung cancer cell line (NCI-H69AR), which expresses the ABCC1 efflux pump protein. Table 5 and Figure 7 revealed that both **7b** and **10e** displayed growth inhibitory activity towards NCI-H69AR cells with IC_50_ values of 16.0 µM. Accordingly, the NCI-H69AR cells were about four-fold less sensitive than A-549 cells, suggesting that these compounds might be subjected to efflux by ABCC1. Furthermore, the H69AR cells manifested a lesser degree of resistance to the reference drug, Sunitinib (1.9-fold less sensitive).

**Table 5.** Cancer cell growth inhibitory activity of compounds **7b**, **10e**, and Sunitinib towards sensitive (A-549) and resistant (NCI-H69AR) cell lines.

	IC_50_ (µM)		
Compound No.	Sensitive A-549	Resistant NCI-H69AR	Fold resistant
**7b**	3.8 ± 0.67	16.0	4.2
**10e**	4.1 ± 0.61	16.6	4.0
Sunitinib	3.1 ± 0.9	5.8 ± 0.52	1.9

## Conclusion

In summary, herein, we report the design and synthesis of different series of hydrazonoindolin-2-ones **3a-e**, **5a-e**, **7a-c**, and **10a-l**. All the prepared hydrazonoindolin-2-ones were examined for their anti-proliferative activity towards three cell lines, namely lung A-549, colon r HT-29, and breast ZR-75 human cancer cell lines using CellTiter-Glo™ Luminescent cell viability assay. Compounds **5b**, **5c**, **7b**, and **10e** were the most potent members with average IC_50_ values of 4.37, 2.53, 2.14, and 4.66 µM, respectively, which are superior to Sunitinib (average IC_50_ = 8.11 µM). The SAR study suggested that replacing the phenyl moiety of compounds **3a-e** with 2-thiazolyl ring, 5-pyrazolyl ring, or its substitution with a lipophilic ester group greatly enhances their anti-proliferative activity. In addition, hybridisation of the indolin-2-one moiety with 4-phenylthiazolyl moiety, compounds **10a-l**, seems to be a prosperous approach. Furthermore, compounds **7b** and **10e** were evaluated for their effects on cell cycle progression and levels of phosphorylated retinoblastoma (Rb) protein in the A-549 human cancer cell line. Moreover, **7b** and **10e** inhibited the cell growth of the multidrug-resistant lung cancer NCI-H69AR cell line with IC_50 _= 16 µM. Finally, the cytotoxic activities of **7b** and **10e** were assessed towards three non-tumorigenic cell lines (Intestine IEC-6, Breast MCF-10A, and Fibroblast Swiss-3t3) where both compounds displayed mean tumor selectivity index (1.6 and 1.8) higher than that of Sunitinib (1.4).
